# Interdiction in the Early Folding of the p53 DNA-Binding Domain Leads to Its Amyloid-Like Misfolding

**DOI:** 10.3390/molecules27154810

**Published:** 2022-07-27

**Authors:** Fernando Bergasa-Caceres, Herschel A. Rabitz

**Affiliations:** Department of Chemistry, Princeton University, Princeton, NJ 08544, USA; hrabitz@princeton.edu

**Keywords:** cancer, prion, folding, pathway, interdiction, peptide

## Abstract

In this article, we investigate two issues: (a) the initial contact formation events along the folding pathway of the DNA-binding domain of the tumor suppressor protein p53 (core p53); and (b) the intermolecular events leading to its conversion into a prion-like form upon incubation with peptide P8(250-257). In the case of (a), the calculations employ the sequential collapse model (SCM) to identify the segments involved in the initial contact formation events that nucleate the folding pathway. The model predicts that there are several possible initial non-local contacts of comparative stability. The most stable of these possible initial contacts involve the protein segments ^159^AMAIY^163^ and ^251^ILTII^255,^ and it is the only native-like contact. Thus, it is predicted to constitute “Nature’s shortcut” to the native structure of the core domain of p53. In the case of issue (b), these findings are then combined with experimental evidence showing that the incubation of the core domain of p53 with peptide P8(250-257), which is equivalent to the native protein segment ^250^PILTIITL^257^, leads to an amyloid conformational transition. It is explained how the SCM predicts that P8(250-257) effectively interdicts in the formation of the most stable possible initial contact and, thereby, disrupts the subsequent normal folding. Interdiction by polymeric P8(250-257) seeds is also studied. It is then hypothesized that enhanced folding through one or several of the less stable contacts could play a role in P8(250-257)-promoted core p53 amyloid misfolding. These findings are compared to previous results obtained for the prion protein. Experiments are proposed to test the hypothesis presented regarding core p53 amyloid misfolding.

## 1. Introduction

Since its discovery in 1979 [1,2,3,4], considerable evidence has accumulated on the critical importance of the tumor suppressor factor p53 in the natural protection mechanisms against cancer development [5,6]. Around 50% of all cancers show altered functionality of p53, frequently through mutation [7,8,9]. Thus, considerable efforts have focused on understanding the natural roles of p53 in protecting against cancer [10,11,12], and also in finding ways to restore its natural activity in cancer patients [13,14]. The most important role of p53 seems to be tumor suppression through the induction of apoptosis cellular programs in response to stress signals [15,16]. Additional tumor suppression-related activities have been discovered, such as metabolic regulation, autophagy and changes in the oxidative state of the cell [17,18,19]. Recently, it has also been found that p53 has further roles in normal physiology and the etiology of other diseases [20]. Thus, the complete biophysical characterization of p53 and its functionalities is an issue of prime importance.

Similar to other transcription factors, the 393 amino acids of p53 make it a multi-domain protein [21,22]. Its five domains include an N-terminal activation domain (residues 1-63), a proline-rich domain (residues 64-93), a DNA-binding domain, which is known as the core domain (i.e., core p53, residues 94-297), a tetramerization domain (residues 298-355), and a C-terminal domain (residues 356-393). The DNA binding of p53 to cellular DNA is carried out by the core domain of p53, and more than 95% of the tumor-inducing mutations of p53 occur in the core p53 [9,23,24]. Furthermore, p53 is a very flexible protein that can attain different closely related native conformations [25,26].

A striking recent discovery has been that under amyloidogenic experimental conditions, p53 and its individual domains undergo amyloid misfolding and aggregation [27,28,29,30,31,32,33]. These promoting factors include: (a) several point mutations that destabilize core p53; (b) denaturing conditions; (c) incubation with specific aggregation-seeding external agents, such as the peptide P8(250-257) resembling the native segment ^250^PILTIITL^257^ [34,35]. The amyloid aggregates of p53 have been shown to display prion-like properties in vivo that could have a direct bearing on the evolution of several tumor types. These properties include: (a) Several types of cancer tissues show abnormal amyloid-like aggregates of misfolded p53 [32]; (b) p53 amyloid formation leads to cellular pro-metastatic gain-of-function [36]; (c) p53 amyloid-fibrils seed misfolding and aggregation when internalized in cells [37], and (d) p53 amyloid formation in cells impairs its transcriptional regulation function [34].

The amyloid misfolding and aggregation of the prion protein is understood to play a key role in triggering several neurodegenerative diseases [38], including Creutzfeldt-Jakob disease in humans [38] and mad cow and scrapie in cows and sheep, respectively [38]. It is likely that similar misfolding and aggregation mechanisms underlie other more widespread human pathologies, such as Parkinson’s disease [39,40], Lewy-body dementia [39,40] and, possibly, Alzheimer’s disease [41]. The existence of a prion-like amyloid misfolding-aggregation behavior for p53 suggests the possibility that there might be substantial molecular commonalities between at least some types of cancer and neurodegenerative diseases. There is, however, still some uncertainty concerning whether the p53 aggregates are infectious in the same way as prion aggregates [42]. The firm establishment of such a common molecular basis for apparently unrelated and widespread diseases could be of major importance, leading to opportunities for the development of related therapeutic strategies [33]. Here, prion or amyloid-like refer only to the misfolding of p53 into amyloid species that can aggregate into higher-order oligomers and fibrils and propagate inside an individual, not to the possible infectious properties of such amyloids.

The purpose of this paper is to: (a) predict the possible initial contact formation events along the folding mechanism of the core domain of P53; and (b) study the effects on the dominant native folding pathway of incubation with peptide P8(250-257). This peptide has been experimentally shown to promote the prion-like transformation of the core domain of P53 upon incubation with peptide P8(250-257), which resembles the native sequence segment 250-257 [34,35]. The calculations will employ the sequential collapse model (SCM) for the protein folding pathways [43,44].

Our calculations predict that there are several possible initial contact formation events that potentially lead to multiple folding pathways. It is also predicted that the more stable possible initial contact (i.e., the dominant contact) involves the protein segment 251-255. This prediction leads naturally within the model to the hypothesis that incubation of the core domain of p53 under denaturing conditions, with P8(250-257), may interdict in the dominant folding pathway, which is a process previously studied for several viral proteins with the SCM [45,46]. The possible interdiction effect arises because P8(250-257) competes with the key interactions defining the dominant early contact formation event. The experimental observation that such incubation produces the transformation of the core domain into a prion-like species is then explained to be naturally understood within the model, as evidence that such a misfolding is likely linked to enhanced folding (i.e., actually protein misfolding) through one or several of the secondary pathways initiated by the less stable contacts. In this paper, these findings will be compared to similar previously obtained results for the misfolding of the prion protein [47]. Possible experiments to test the hypothesis for p53 are discussed.

## 2. Results

### 2.1. The Physical Basis of Non-Local Early Contact Formation in the SCM

**Figure 1:**
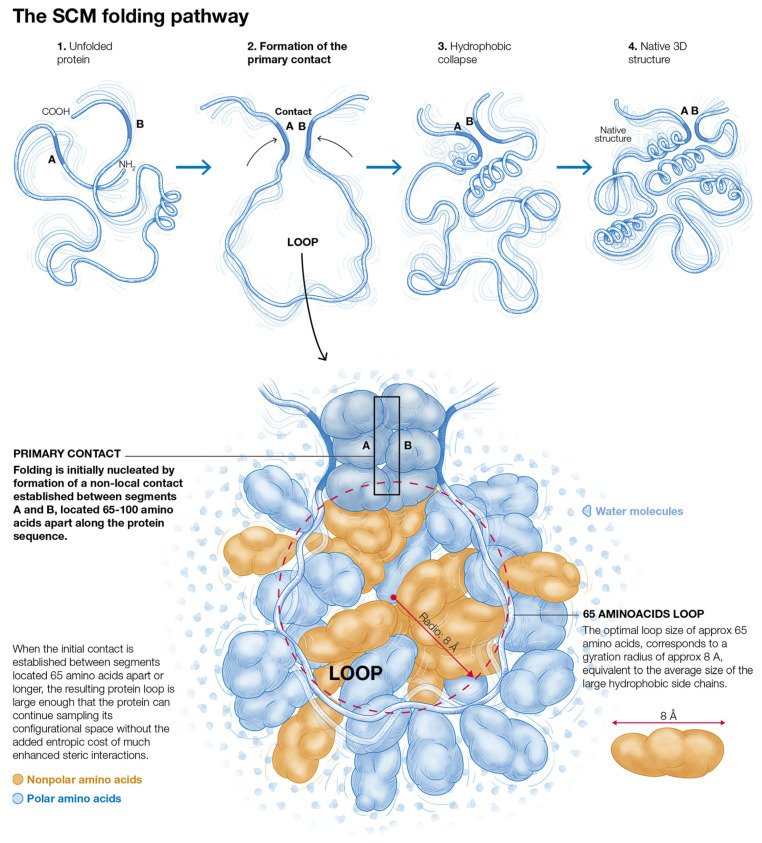
The physical basis for the formation of early non-local contacts in the SCM. The segments forming the contact are labeled A and B.

The physical basis of the SCM and its most up-to-date formulation has been recently explained in detail [44], and the associated calculation methodology is summarized in the Methods section of this paper. Here, a brief introduction to the main concepts is presented that are relevant to the issues investigated in the present paper. The SCM considers early non-local contacts based on the entropy of formation of the resultant protein loops in the unfolded state and the hydrophobic stabilization energy of the protein segments that define the contacts. The SCM has successfully predicted many of the observed features of the protein folding pathways at a low resolution [44]. Within the SCM, the folding of proteins with more than ~100 amino acids is nucleated by the formation of a specific early non-local contact, called the primary contact, which defines the earliest folding phase. The primary contacts between two protein segments centered at the residues i and j, separated by a distance along the protein sequence n_ij_, form references to an optimal distance n_op_ such that n_ij_ ≥ n_op_ ≈ 65 amino acids, where the actual contact at n_ij_ is determined by the excluded volume-related entropic consequences of forming early protein loops [44]. The physical basis for the formation of the early non-local contacts in the SCM is illustrated in Figure 1.

For a given protein sequence, there might be several viable primary contacts that nucleate parallel folding pathways [44]. As at most, only a few simultaneous primary contacts can be established in proteins of length n ≥ n_op_, while most of the tertiary structure contacts will still be defined by contacts at a shorter range established in later folding phases [44]. The formation of the primary contact in the SCM defines the primary loop, which subsequently collapses through two-state kinetics [44]. The nucleation by an early primary contact has been referred to within the model as “Nature’s shortcut to protein folding” [44]. The short-range contacts established in later folding phases are defined by fluctuating shorter loops called minimal loops in the model, which are expected to be of length n_min_ ~15 amino acids [43]. Thus, within the model, the observed persistence length of the loops in a native protein is expected to be significantly lower than n_op_ and closer to n_min,_ in agreement with the experimental observations [48]. It is important to bear in mind, however, that the SCM is concerned with the optimal sizes of loops in the fluctuating unfolded chain rather than with the topology of the fully folded protein. The final length of the topological elements of the 3D structure can vary from their open-chain seeding loops, as contacts in the folded structure are refined by optimal packing, secondary structure formation and the establishment of all the relevant interactions [49]. Because proteins longer than ~100 amino acids do not generally undergo a complete two-state collapse [44,50] but rather fold through multi-step pathways, consistent and simple physical reasoning implies that there is a limit to the size of the primary loop (i.e., ~100 amino acids) that can successfully lead to the native SCM folding pathway.

The concept of folding nucleation by non-local contacts is not exclusive to the SCM, having arisen earlier in the context of the diffusion-collision model [51], the loop hypothesis [52] and the energy landscape picture [53]. Furthermore, it has appeared in simulations of the transition state of two-state folding proteins [54]. Protein topology has been considered an essential element of folding mechanisms in a number of theoretical efforts [48,55,56,57,58]. The particular feature of the SCM is that the early non-local contacts are highly specific, as in the loop hypothesis [52], and the SCM provides a general method to determine their location at specific distances along the primary sequence [43,44].

### 2.2. Primary Contacts of Core p53

Following the methodology employed in the SCM before [43,44], which is described in detail in the Methods section, our calculations searched for the most stable possible hydrophobic contacts between pairs of 5-amino acid segments centered at amino acids i and j, located at a distance n_ij_ along the sequence such that 65 ≤ n_ij_ ≤ 100 amino acids. The calculations employ the primary sequence and experimental hydrophobicity values to determine the location of the possible primary contacts. The results were seen to be robust when extending the segment lengths up to seven amino acids. Here, we will keep the analysis focused on the 5-amino acid segment results for the purpose of consistency and the ability to compare with the previous work within the SCM. The predicted primary contacts and their stabilities are listed in Table 1. The stabilities in the table correspond to the contacts as formed in the earliest folding phase, not on the fully folded structure.

**Figure 2:**
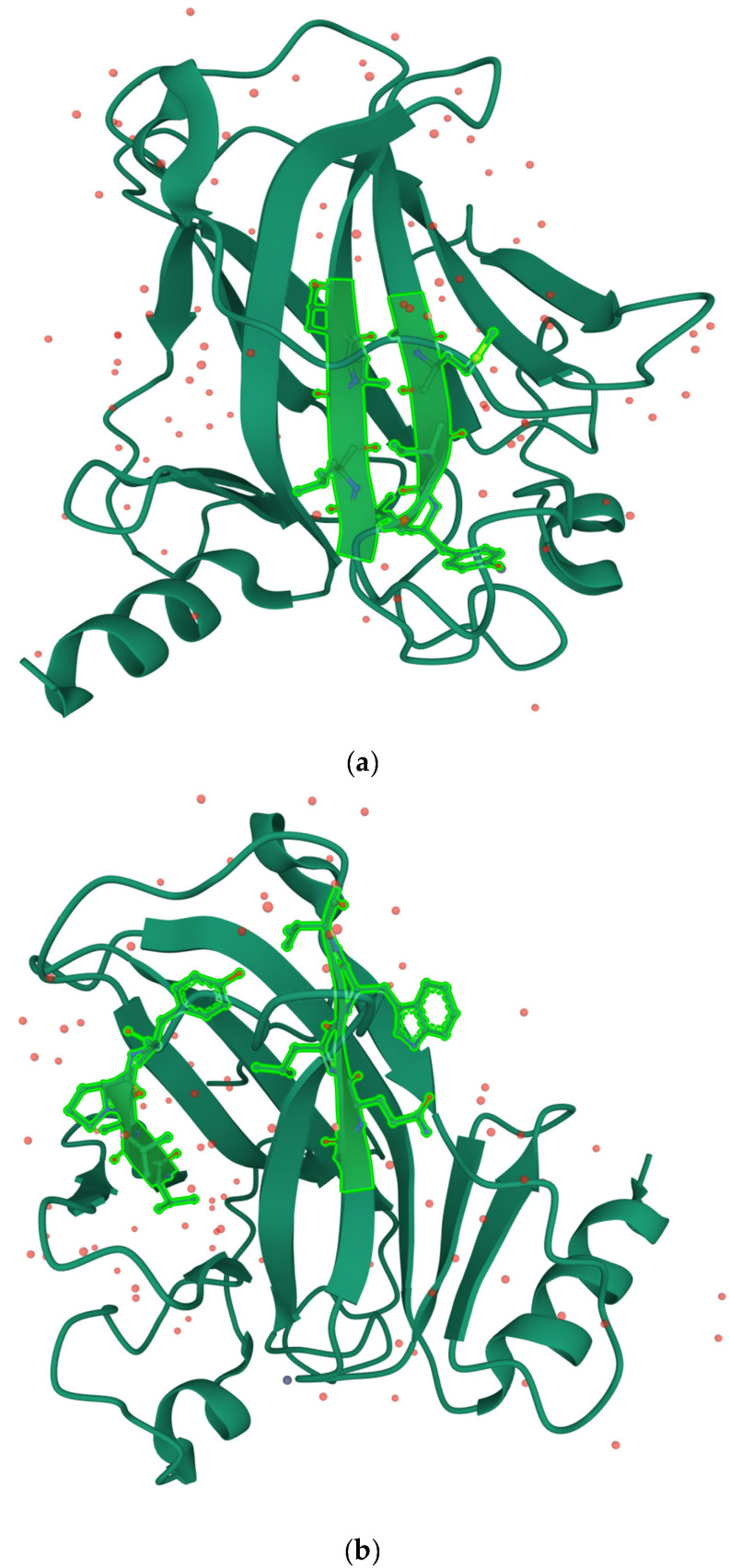
(**a**) The best possible primary contact, C1, on the structure of core p53 (PDB 2OCJ); (**b**) contact C2 is on the same structure. As can be observed in the figures, C1 is a good contact on the 3D structure, while C2 does not, as the side chains of the two segments that define it are not close in the folded structure. The figures were prepared using Mol* [60].

The most stable primary contact, C1, is predicted to form between the segments ^159^AMAIV^163^ and ^251^ILTII^255^, with a predicted stability of ΔG_cont_(C1) ≈ −8.9 *k*T. It is a native contact on the 3D structure [59], as shown in Figure 2.

The second best possible primary contact, C2, is established between the segments ^143^VQLWV^147^ and ^216^VVVPY^220^, with a contact stability of ΔG_cont_(C2) ≈ −8.2 *k*T. Contact C2 is not a good match for the 3D structure, as shown in Figure 2. There are two additional possible non-native contacts, C3 and C4, both with a lower stability of ΔG_cont_(C3) = ΔG_cont_(C4) ≈ −7.8 *k*T. All the other possible primary contacts are more than ~3 *k*T less stable than the dominant one and non-native on the tertiary structure, with corresponding populations of more than ~one order of magnitude smaller than that of the best contact, and we have not included them in the analysis. The issue of the multiplicity of the possible primary contacts in the SCM has been considered in previous work [44]. Because no major rearrangements of the protein core are expected post-collapse, it is generally assumed within the model that the primary contacts which are non-native in the 3D folded structure likely do not correspond to the native pathways leading to the functional folded structure [44]. In all the naturally folding proteins studied to date within the SCM, it has been observed that the most stable primary contact is native-like [44].

On the basis of the above results, it is reasonable within the SCM to expect that the contact C1 nucleates the native folding pathways for core p53. Thus, C1 constitutes “Nature’s shortcut” to the folding of core p53 [44]. On the other hand, contacts C2, C3 and C4 probably represent non-native-initial contacts that must break up in order for core p53 to enter the native folding pathway nucleated by C1. The nucleation of native folding by the non-native primary contacts would imply a considerable later rearrangement of the protein core, which is entropically unfavorable [61].

### 2.3. Comparison of the Predicted Primary Contact Populations with Experimental Data on Core p53 Folding

If the folding of core p53 proceeds through the four predicted possible nucleation events, it is reasonable to expect that each of the four corresponding folding channels must involve a fraction of the folding proteins that are equivalent to the initial relative population of the corresponding primary contact. Based on their relative stabilities, the predicted relative populations of contacts C1, C2, C3 and C4 are, respectively, 46% for C1, 23% for C2 and 15% for both C3 and C4. From a kinetic point of view, as ΔG_cont_(C3) = ΔG_cont_(C4), the break-up of both contacts should take place within similar time scales, and thus, in a low-resolution experiment, they would probably appear as a single channel traversed by the combined populations of C3 and C4, with an apparent population of ~31%. The uncertainty in the energy values ΔG_cont_(C) accounts for the single standard deviation confidence intervals of ~[+7%,−7%] for a variation in the population associated with C1, [−6%,+8%] for C2, and [−4%,+6%] for each of C3 and C4.

Experimental evidence exists for core p53 that supports the existence of three kinetically distinct folding channels a, b, and a less well-resolved channel c, with ~50% of the native population folding through a and ~25% through each of b and c [62,63]. The predicted population of each native contact does not necessarily precisely reflect the overall population of molecules fully folding through each folding pathway, as there might be differences in the folding kinetics arising, for example, from the break-up of the non-native primary contacts [63]. Within the SCM, however, it is reasonable to assume that the intermediates nucleated by the four primary contacts will undergo a hydrophobic collapse with broadly similar kinetics once the native primary contact is established [64]. Thus, taking into account their equivalent stabilization energy, C3 and C4 should be difficult to resolve in separate channels in standard kinetic experiments. However, it is fair to say that the populations of the four early native-like intermediates predicted here correlate well with the experimental observations concerning the populations of molecules traversing each of the three folding channels detected experimentally. C1 would nucleate channel a with a population of ~46%, C2 represents channel 2 with a population of ~23%, and C3 and C4 add up to a less resolved channel c with a population of ~31%. Because of the lack of single-residue resolution in the available experimental data, this correlation should be considered as just a consistency test of the SCM predictions.

### 2.4. Interdiction of the Dominant Folding Pathway through Competition of Peptide P8(250-257) with the Formation of Primary Contact C1

**Figure 3:**
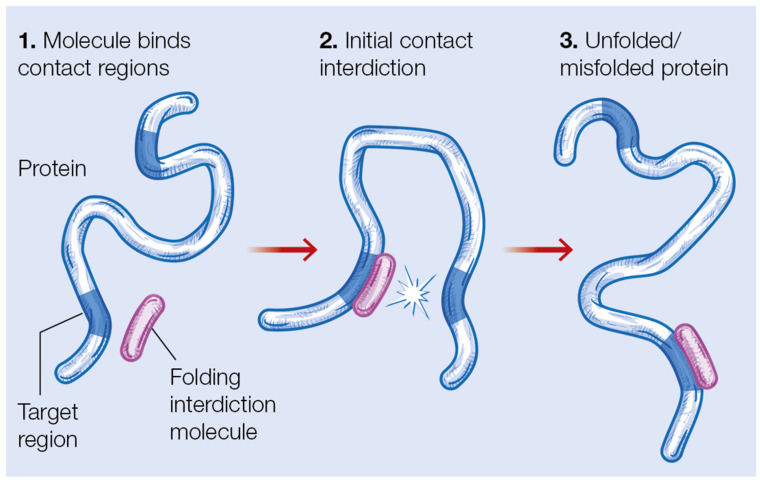
Protein folding interdiction, based on the identification of a FITR target.

In recent work, we proposed that the SCM’s primary contact predictions provide natural targets for folding interdicting peptide drugs, which are aimed to treat viral diseases, such as SARS-CoV-2, Ebola and influenza A [45,46] by blocking the initial folding steps of specific viral proteins. Such peptide drugs could be designed by employing, as templates, the segments naturally involved in the primary contact, where one of the segments, S_1_, would be the basis of the folding interdicting peptide (FIP), and the other segment, S_2_, would constitute the folding interdiction target region (FITR), as described in Figure 3. In such an interdiction mechanism, the FIP would compete with S_1_ to bind to S_2_, leading to a decrease in the number of proteins folding through the pathway initiated by the contact S_1_-S_2_.

It is interesting to investigate whether the FITR concept could be applied to core p53, such that specific peptide drugs can be designed to modulate its folding dynamics and, potentially, its physiological activity. In particular, it would be of considerable importance to determine, through selective interdiction of the possible folding pathways, whether any or several of them are involved in the prion-like transition of p53. If that was the case, it might be possible to design specific peptide drugs aiming to interdict the misfolding pathways, thus maximizing the population of natively folded p53. Below, we will explain that such a folding interdiction experiment has already been partially carried out for the core domain of p53 [34,35].

Experiments have shown that the incubation of the core domain of p53 with peptide P8(250-257), defined by the native sequence ^250^PILTIITL^257^, induces amyloid-like aggregation [34,35]. Peptide P8(250-257) is just the segment ^251^ILTII^255^ predicted to form the native primary contact C1 with segment ^159^AMAIY^163^, plus residues P250, T256 and L257, amino acids that are non-polar or hydrophobic and, thus, will tend to promote further contact formation. Thus, within the current model, the prediction is clear that the intermolecular effect of P8(250-257) on the folding of core p53 is the formation of the contact between P8(250-257) and the segment ^159^AMAIY^163^, leading to interdiction in the native folding pathway of core p53.

Experimental evidence also shows that P8(250-257) tends to form β-sheet-rich aggregates by itself [35], suggesting that these aggregates may act as a seed for the prion-like misfolding of p53 [35]. Most of segment ^250^PILTIITL^257^ defines a strand of a β-sheet in the native structure of core p53, in close interaction with the strands ^264^LLGRNSFEVRV^274^ and ^156^RVRAMAIY^163^ that includes the segment ^159^AMAIY^163^ which also nucleates C1 [59]. Thus, it is reasonable to hypothesize that the ß-sheet-rich aggregates of P8(250-257) can also interdict in the formation of the primary contact C1 by interacting with the segments that define C1. This result also suggests that P8(250-257) alone might be able to interdict in the formation of C1, not just by its attachment to segment ^159^AMAIY^163^, but also to segment ^251^ILTII^255^ itself, thus enhancing the interdiction effect. Only more detailed experimental and theoretical studies can determine which of the possible interdiction modes described here, if any, is dominant in triggering the experimentally observed misfolding effects.

### 2.5. Possible Coincidence of the p53 and Prion Protein Misfolding Mechanisms

If interdiction in the formation of the dominant native-like primary contact C1 triggers the misfolding and subsequent aggregation of core p53 when incubated with peptide P8(250-257), a relevant question is by what molecular mechanisms interdiction might lead to misfolding. It is important to keep in mind that there might be more than one mechanism leading to misfolding [65]. For the neuropathogenic protein, for example, different mutations lead to distinct amyloid formation and aggregation dynamics [65,66,67]. In the case of core p53, several mutations with no obvious connection to the interdiction mechanism described here (i.e., involving the mutations outside the predicted primary contacts), such as R175H, R249S, R273H, C242S and R248Q, lead to amyloid formation under the appropriate conditions [68,69].

In recent work, we proposed that a few specific mutation-related misfolding events of the murine prion protein *m*PrP(90-231) were related to the protein traversing through a folding pathway nucleated by a primary contact of lesser stability than the dominant contact [47]. Furthermore, we showed that several known pathogenic mutations have the effect of increasing the relative population of prion proteins entering the secondary pathways defined by the less stable contacts [47]. It then becomes an interesting question of whether a similar mechanism could be at play in the interdiction-triggered prion-like conversion of core p53.

**Figure 4:**
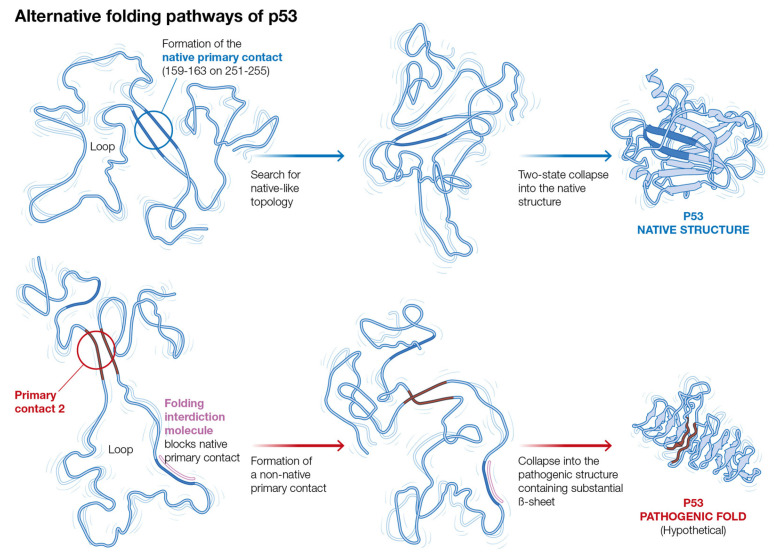
Hypothetical misfolding mechanism of core p53 triggered by interdiction with peptide P8(250-257).

Within the current model, the experimental observation that the incubation of core p53 with peptide P8(250-257) leads to its conversion into a prion-like species, combined with our previous results for the prion protein, suggests that one or several of the less stable contacts might nucleate a folding pathway, thus leading to misfolded core p53. Then, interdiction in the dominant native pathway, with a concomitant reduction in the population of molecules folding through the native pathway, could lead to increased folding through the less stable primary contacts, including the pathogenic pathway/s leading to abnormal levels of misfolded p53 that aggregate into amyloid-like inclusions. This hypothetical mechanism is shown in Figure 4.

At the current level of the analysis, it is impossible to predict unequivocally whether such a mechanism is in play, and, in the absence of detailed experimental evidence, it should be considered as a hypothesis. Another possibility is that interdiction in the formation of the dominant primary contact by either monomeric or aggregated P8(250-257) leads to a direct amyloid transition. A possible experiment to test the secondary folding channel hypothesis could be carried out by interdicting in the formation of the non-native contacts under amyloidogenic conditions. If the prion-like transition of core p53 proceeds through the initial formation of any of the non-native contacts C2–C4, interdiction in the formation of that contact should inhibit it. Thus, for example, if the amyloid transition was nucleated by the formation of C2, the incubation of core p53 with the peptides derived from either ^143^VQLWV^147^ or ^216^VVVPY^220^ should diminish the formation of prion-like structures under amyloidogenic conditions. There is considerable interest in the investigation of therapeutic drugs that might retrieve the active conformation of p53 [70] and, more specifically, that interfere with the formation and effects of the p53 aggregates [71]. The results presented here could potentially open a new avenue towards this goal. However, as discussed above, there might be more than one mechanism leading to an amyloidogenic transition, and the set-up for an experiment such as the one described above, leading to a strong clear-cut result, is likely to be a complex undertaking.

Finally, it is important to point out that the aggregation mechanisms in vivo can be decisively influenced by additional factors, such as pH and metal ion concentrations [72] and other proteins [73]. Thus, the complete validation of the biological relevance of the mechanism for p53 aggregation proposed here would require in vivo testing, probably employing the full range of existing testing techniques [74].

## 3. Conclusions

In this paper, we presented the theoretical predictions for the earliest folding events of the core domain of p53. Several possible initial contact-forming events were identified, potentially leading to a multiplicity of folding pathways. It was also explained that the experimentally observed prion-like transition of the core domain of p53 upon incubation in the denaturing conditions with peptide P8(250-257) could be understood within the SCM through a folding interdiction mechanism, as proposed in earlier work. It was explained how additional experiments could possibly confirm this hypothesis and open a new path to the design of peptide drugs that are able to modulate p53 folding dynamics.

## 4. Methods: Determination of the Primary Contact in the SCM Model

The physical basis of the SCM and its most up-to-date formulation has been recently explained in full detail [44]. Here, only a brief summary of the methodology to determine the primary contact is presented.

Based on the model presented in the previous sections, whether there is a non-local contact in an otherwise unfolded state is dependent upon the stability of the potential contact candidates at the loop lengths of n ≥ n_op_ amino acids. In the SCM, the stability of a contact formed by the number n_cont_ of amino acids, ΔG_contact_(n_cont_, n_loop_), can be written as:

ΔG_contact_(n_cont_, n_loop_) ≈ ΔG_int,H_(n_cont_) + ΔG_loop_(n_loop_) + ΔG_cont,S_(n_cont_) (1)

Here, ΔG_loop_ represents the entropic free energy cost of the loop, as discussed in Section 2.1. The term ∆G_int,H_ denotes all the enthalpic interactions that help stabilize the contact, which possibly includes the hydrophobic interactions, van der Waals interactions, hydrogen bonds, disulfide bonds and salt bridges [49], and its value satisfies ∆Gint < 0. The term ΔG_cont,S_ > 0 represents the entropic cost of constraining the side chains of the amino acids, defining the contact as such that the contact is stable and it opposes contact formation. A segment-specific determination of the value ΔG_cont,S_(n_cont_) for a given contact would require detailed molecular dynamics techniques. However, a heuristic estimate can be made from earlier work within the SCM, which showed that the average entropic cost of folding per amino acid for a sample of thirteen proteins was ΔG_folding/residue,S_ ≈ 0.85 *k*T/residue [75], and the maximum was ΔG_folding/residue,S_ ≈ 1.09 *k*T/residue. As these are estimates for the entropic cost for folding per residue of the complete proteins that include highly buried as well as flexible exposed regions, it is then reasonable to expect that the entropic cost of a contact-forming region must be closer to the highest calculated values or ΔG_folding/residue,S_. Here, we will assume that ΔG_contact,S_(n_contact_) for a contact including the n_cont_ amino acids is approximately ΔG_folding/residue,S_, determined by the number of residues defining the contact, such that ΔG_cont,S_(n_cont_) ≈ 1.09 n_cont_. This result is clearly an approximation, but it suffices for establishing a cutoff in the number of possible contacts, which is consistent with the available structural data.

Hydrophobic interactions are well understood to constitute the main driving force of the folding process [49]. Other interactions, such as hydrogen bonds, are weaker [76], or like disulfide bonds and salt bridges, form later along the folding pathway [49]. Thus, for an early contact that forms from the unfolded state, we can take ∆G_int_(n_op_) ≈ ∆G_hyd_(n_op_), where ∆G_hyd_(n_op_) represents the stabilizing effect of hydrophobicity in the early contacts, and Equation (1) can be written as

ΔG_contact_(n_cont_, n_loop_) ≈ ΔG_hyd_(n_cont_) + ΔG_loop_(n_loop_) _+_ ΔG_contact,S_(n_contact_) (2)

Since the hydrophobic stabilization energy of the contact ΔG_hyd_ is determined by the hydrophobicity of the segments involved, the hydrophobicity values h_k_ are obtained from the Fauchere–Pliska scale [77] and assigned to each residue in accordance with previous calculations within the SCM.

Because the amino acid side chains are significantly larger than the typical peptide bond length, early contacts between two hydrophobic amino acids will inherently involve segments, including several amino acids, adjacent to the initial contact. The stability of this early hydrophobic contact will determine where the folding process is initiated. This picture is not unlike the zapping model of Dill and collaborators [78] and includes a well-defined nucleation step, as expected in most protein folding models [79]. Here, the typical early contact segment size will be taken to be ~5 amino acids, in line with previous calculations within the SCM. The 5-amino acid window size is based on the geometric considerations underlying the SCM: With an average effective fluctuating width of the unfolded protein chain of w ~2 ʆ(n) ≈ 15.8 Å and a peptide bond length of 3.5 Å, the minimum number n_cont_ of amino acids that can define a contact in the open fluctuating chain should be n_cont_ ~ int[2 ʆ(n)/3.5] = 5 amino acids. The results for the location of the most stable primary contact were seen to be robust to the employment of up to seven amino acid windows, while some deviations were observed when the window was reduced to four amino acids. In practice, within the SCM, the hydrophobicity h_k_ of each residue is added over a segment contact window of five amino acids centered at residue i, resulting in a segment hydrophobicity h_i_,_5_ (a value of ~0.45 is equivalent to a change in the energy of *k*T, with the margin of error being ~0.1 *k*T [77]).

In order to determine the best contact, the h_i,5_ values of a segment centered at residue i are added to the h_j_ value of a segment centered at residue j, located at a distance n_ij_ at least n_op_ amino acids apart along the sequence, and no longer than the maximum primary loop length of ~100 amino acids, to give a contact stability of:

ΔG_cont_(n_cont_, n_loop_) ≈ *k*T [−(h_i,5_ + h_j,5_)/0.45 + 3/2 ln n_ij_ + 10.9] 100 ≥ n_ij_ ≥ 65 (3)

Finally, the relatively simple algorithm presented here has sufficed to successfully predict primary contacts within the SCM so far. However, specific issues critical for drug design, such as identifying the optimal interdiction molecules and fully characterizing their interactions, will probably require a more sophisticated approach, including state-of-the-art molecular dynamics [80].

## Figures and Tables

**Table 1:** Possible primary contacts for the core domain of core p53, their stabilization energy (*k*T), and their location in the native 3D structure.

	Contact	Stability	3D Structure
C1	^159^AMAIY^163^ on ^251^ILTII^255^	8.9 ± 0.3	Native
C2	^143^VQLWV^147^ on ^216^VVVPY^220^	8.2 ± 0.4	Non-Native
C3	^143^VQLWV^147^ on ^234^YNYMC^238^	7.8 ± 0.4	Non-Native
C4	^133^MFCQL^137^ on ^216^VVVPY^220^	7.8 ± 0.4	Non-native

## Data Availability

Not applicable.

## Abbreviations

SCM, Sequential Collapse Model.
